# Molecular phylogeny reveals distinct evolutionary lineages of the banded krait, *Bungarus* *fasciatus* (Squamata, Elapidae) in Asia

**DOI:** 10.1038/s41598-023-28241-8

**Published:** 2023-02-04

**Authors:** Lal Biakzuala, Hmar T. Lalremsanga, Vishal Santra, Arindam Dhara, Molla T. Ahmed, Ziniya B. Mallick, Sourish Kuttalam, A. A. Thasun Amarasinghe, Anita Malhotra

**Affiliations:** 1 Developmental Biology and Herpetology Laboratory, Department of Zoology, Mizoram University, Aizawl, Mizoram 796004 India; 2 Society for Nature Conservation, Research and Community Engagement, Nalikul, Hooghly, West Bengal 712407 India; 3 Captive and Field Herpetology, 13 Hirfron, Anglesey, LL65 1YU Wales UK; 4 Molecular Ecology and Evolution at Bangor, School of Natural Sciences, College of Environmental Sciences and Engineering, Environment Centre Wales, Bangor University, Bangor, LL57 2UW UK; 5 Department of Biology, Faculty of Mathematics and Natural Sciences, Universitas Indonesia, Kampus UI, Depok, 16424 Indonesia

**Keywords:** Herpetology, Phylogenetics, Taxonomy

## Abstract

The banded krait, *Bungarus* *fasciatus* is a widespread elapid snake, likely to comprise several distinct species in different geographic regions of Asia. Therefore, based on molecular phylogenetics and comparative morphology data, we present an overview of the systematic composition of the species to delimit potential biogeographic boundaries. Our phylogenetic analyses, based on four mitochondrial genes, reveal the existence of at least three evolutionary lineages within *B.* *fasciatus*, corresponding to Indo-Myanmar, Sundaic and eastern Asian lineages. We are convinced that there are at least three taxonomic entities within the nomen *B.* *fasciatus* and restrict the distribution of *B.* *fasciatus* sensu stricto to the Indo-Myanmar region. We also provide additional natural history data of the taxon from eastern India. Finally, we advocate further studies to establish the degree of reproductive isolation among these diverging evolutionary lineages and to reassess the systematic status of this species complex especially the Sundaic and eastern Asian lineages.

## Introduction

Aside from its taxonomical importance, recognition and ascertainment of independently evolving lineages is crucial for understanding the evolutionary processes affecting the origin of population structure and species diversification^1^. Because of the growing availability of genetic methods for species delineation^2^, numerous studies have uncovered cryptic diversity within the widespread vertebrate species including in tropical and sub-tropical Asia; for instance, among fishes^3–5^, amphibians^6–8^, birds^9–11^, and mammals^12–14^. Moreover, recent phylogeographical and molecular studies have refined our understanding of cryptic speciation across biogeographic boundaries or within biogeographic regions^15,16^, and even propounded the suitability of reptiles in particular as biogeographic indicators^17,18^. Recent studies focussing on widespread reptilian species have also established the existence of previously unnoticed cryptic diversity, including in lizards^19–22^ and snakes^23–30^.

*Bungarus* Daudin, 1803, collectively known as kraits, are venomous elapid snakes which inhabit the Asian subcontinent^31^. Most of the nominal *Bungarus* species are poorly understood. However, recent study on the diversification and evolution of elapid snakes have highlighted that the diversification of kraits occurred around 30–25 million years ago, and are close relatives of other Australasian elapid genera and sea snakes^32^. *Bungarus* *fasciatus* (Schneider, 1801), commonly known as the banded krait, is a nocturnal and conspicuous krait that grows up to 2,250 mm in total length and is morphologically characterized by its yellow (or cream) and black banded body^33^. It occurs in various habitat types such as primary forests, agricultural lands as well as domestic gardens up to 2,300 m above sea level^33,34^. So far, *B.* *fasciatus* has been reported from eastern India, Nepal, Bhutan, Bangladesh, and Myanmar, extending southwards through Thailand, Malaysia and Singapore into the Indonesian archipelago, and eastwards through Laos, Vietnam and China^35,36^. The species is currently listed as a Least Concern (LC) species in the IUCN Red List^35^. Despite its wide distribution, studies have so far been conducted mainly on its potential medical significance^37^, ecological importance^38,39^, or characterization of venom^40–45^.

Although there are no studies specifically on the molecular systematics of this species, several previous studies have highlighted intra-specific or geographical variability based on genetic barcoding^46–48^. Accurate species delimitation is crucial in view of the variability in snake venom composition^49^ and its potential effects on antivenom efficacy^50^. Most of the existing taxonomic and systematic literature on *Bungarus* have apparently overlooked the intraspecific diversity of *B.* *fasciatus*^51–58^. Therefore, in this study we fill in the inherent knowledge gaps by providing comparative morphological evidence and molecular phylogeny based on four mitochondrial genes (COI, CYTB, ND4 and 16S rRNA) based on sequences from east and northeast India, Indochina, and the Greater Sunda islands. Moreover, given the minimal knowledge on the natural history, reproductive behaviour, and ecology, which are important for assessing the population status of the species^34,59^, we also provide natural history data for the populations of *B.* *fasciatus* from India.

## Materials and methods

### Sampling

For this study, we collected both morphological and genetic data for *Bungarus* *fasciatus*, which we compared to publicly available or unpublished data. We collected morphological data for the *B.* *fasciatus* population represented by 15 specimens from northeastern India between the years 2007–2022. We surveyed during the day and night, collected individuals by hand, and euthanized them with MS-222 following the standard procedure^60^ in compliance with the American Veterinary Medical Association (AMVA) guidelines and approved by the Institutional Animal Ethics Committee (IAEC) (Permission No. MZU-IAEC/2018/12). We then fixed the specimens in 10% buffered formalin solution overnight, prior to their storage in 70% ethanol. We preserved liver tissue samples for DNA analysis in 95% ethanol, which were stored at −20 °C. Vouchered specimens were deposited at the Departmental Museum of Zoology, Mizoram University (MZMU). Additional blood samples from the caudal sinus were collected from the West Bengal (WB) populations and preserved in EDTA-Tris buffer; these specimens were subsequently released after taking necessary scale counts. Our study is reported in accordance with the ARRIVE 2.0 guidelines (Animal Research: Reporting of In Vivo Experiments)^61^. The distribution map was prepared using QGIS v3.16.2 and the digital elevation model (DEM) was downloaded from Open Topography (https://opentopography.org/).

### DNA extraction, amplification and molecular analyses

Liver tissue or blood was used to extract genomic DNA using DNeasy (Qiagen™) blood and tissue kits following the manufacturer’s instructions. Fragments of four mitochondrial (mt) markers (16S, COI, ND4 and CYTB) were amplified in a 20 μL reaction volume, containing 1X DreamTaq PCR Buffer, 2.5 mM MgCl_2_, 0.25 mM dNTPs, 0.2 pM of each gene primer pair, approximately 3.0 ng of extracted DNA, and 1 U of Taq polymerase. A negative control with reagent grade water instead of DNA template was always included. Target mt gene sequences were amplified using the thermal profiles and primers given in Supplementary Table S1. PCR products were checked using gel electrophoresis on a 1.5% agarose gel containing ethidium bromide. The PCR products were cleaned using ThermoFisher ExoSAP-IT PCR product cleanup reagent and subsequently sequenced using the Sanger dideoxy method using the ABI 3730xl DNA Analyzer at Barcode BioSciences, Bangalore, India. The generated partial gene sequences were deposited on the NCBI repository (GenBank accession numbers are given in Supplementary Table S2). In this study, a total of one COI, six 16S, six ND4, and nine CYTB sequences were generated and were combined with published sequences of *B.* *fasciatus* obtained from the NCBI database; database sequences of *B.* *caeruleus*, *B.* *candidus*, *B.* *ceylonicus*, *B.* *sindanus*, and *B.* *multicinctus* were used as outgroups. The four mt gene alignments were concatenated in SequenceMatrix^62^. Using the CYTB dataset, the uncorrected p-distance was estimated in MEGA X using the complete deletion option for the treatment of gaps/missing data^63^. Prior to the Bayesian analysis, PartitionFinder v2.1^64^ was utilized to search the best partitioning schemes and the best fitting model through Bayesian Information Criterion (BIC) (Supplementary Table S3). Bayesian phylogeny (BI) was reconstructed using the selected models in Mr.Bayes v3.2.5^65^. The MCMC was run with four chains (one cold and three hot chains) for 20 million generations and sampled every 5000 generations. Tracer v1.7^66^ was used to check the convergence of likelihood and the burn-in cut-off. The diagnosis of topological convergence and MCMC and mixing of chains was done in R-Studio^67^ using the package, R We There Yet (RWTY)^68^. The BI tree was further illustrated using web-based tree annotator iTOL software v5^69^. The Maximum Likelihood (ML) tree was reconstructed in IQ-TREE^70^ using 10,000 Ultrafast Bootstrap (UFB)^71^ based on the dataset partitioned by codon positions with the most appropriate model selected for each partition using ModelFinder^72^ integrated in IQ-TREE^70^. The CYTB dataset, partitioned by codon, was utilized for performing BI and ML based Poisson Tree Processes (PTP) species delineation analyses^73^ implemented in iTaxoTools v0.1^74^. For the input file of PTP, a non-ultrametric tree was produced in IQ-TREE^70^ with 10,000 UFB replicates^71^ using the models selected for CYTB partitions. Only the CYTB dataset was selected for the species delimitation analysis as it contains more samples from different geographical regions compared to the other three genes.

### Morphology

We obtained morphometric (mensural and meristic) data for species comparisons, and distribution data from examined specimens (Java (JV), Mizoram (MZ) and WB) and published literature^54,75–77^. We measured the following characters to the nearest millimetre with a Mitutoyo digital caliper and Leica M50 (Leica Microsystems Inc.) dissecting microscopes: eye diameter (ED, horizontal diameter of orbit); eye–nostril length (EN, distance between anteriormost point of eye and middle of nostril); snout length (ES, distance between anteriormost point of eye and snout); head length (HL, distance between posterior edge of mandible and tip of snout); head width (HW, maximum width of head); snout–vent length (SVL, measured from tip of snout to anterior margin of vent); tail length (TaL, measured from anterior margin of vent to tail tip). Meristic characters were taken as follows: supralabials (SL) and infralabials (IL) (first labial scale to last labial scale bordering gape); dorsal scale rows (DSR, counted around the body from one side of ventrals to the other in three positions, on one head length behind neck, at midbody and at one head length prior to cloacal plate); when counting the number of ventral scales (Ve), we scored values according to the method described by Dowling^78^. We counted subcaudal scales (Sc) from the first subcaudal scale meeting its opposite to the scale before the tip of the tail, the terminal scute is excluded when counting. Sex of the specimens was identified by examining everted hemipenes or by ventral tail dissection. We evaluated the relative size of the nuchal band, the number of the black cross bands of each individual. The number of cross bands on the body (BB) were counted from the first band posterior to the nuchal band on the nape up to the level of cloaca, the count on the tail from the level of cloaca to the tip of tail (BT), and number of vertebral scales covering the nuchal band (NBW). In addition, the number of vertebral scales covering the first cross band is also considered a reliable character for adult individuals. Values for bilateral head characters are given in left/right order. We followed Keogh^79^ for hemipenial terminology, and the extent of inverted hemipenis in terms of percentage of subcaudal scales (HpR).

### Statistical analyses

The morphological information was obtained from three different populations examined by us: recent and long-term preserved specimens from JV in Indonesia (*n* = 15), live specimens from WB (*n* = 8) and live, recent and long-term preserved specimens from MZ (*n* = 15) states in India. Before performing any further analyses, the meristic data were standardized to zero mean and unit standard deviation to avoid potential bias due to difference in the range of measurement among variables; for mensural data, the combination of characters with the highest R-squared score obtained through linear regression was selected as the best log transformation model to make linear relationship with body size. Since we do not have gender information from the WB population, the meristics of the remaining populations (JV and MZ) were first tested using separate one-way analysis of variance (ANOVA) using sex and locality as factors along with Levene’s test^80^ to test the homogeneity of variances; if the assumption of homoscedascity was violated, Brown-Forsythe test^81^ was utilised as an alternative approach. For mensurals (TaL, HL, and HW), a two-way analysis of covariance (ANCOVA) was carried out with snout-vent length (SVL) as a covariate. The meristic variables identified with no sexual dimorphism were utilised for multiple comparison among the three populations by pooling sexes using one-way ANOVA using locality as a factor, and post-hoc was performed with applying Bonferroni correction. In addition, a potential observer difference was screened by repeating measurements on the same specimens and then tested using one-way ANCOVA. The variable characters among lineages identified through the univariate analyses were utilized further for Principal Component Analysis (PCA) to visualize the clustering of the different populations. The correlation matrices between all pairs of the morphological variables, variance explained by each eigenvalue as well as the correlations of each variable to the first two components are explored. Specimens with missing characters were excluded in the multivariate analysis. Statistical analyses were performed using the SPSS v.25.0 statistical package (Armonk, NY: IBM Corp.).

## Results

### Phylogenetic relationship

**Figure 1:**
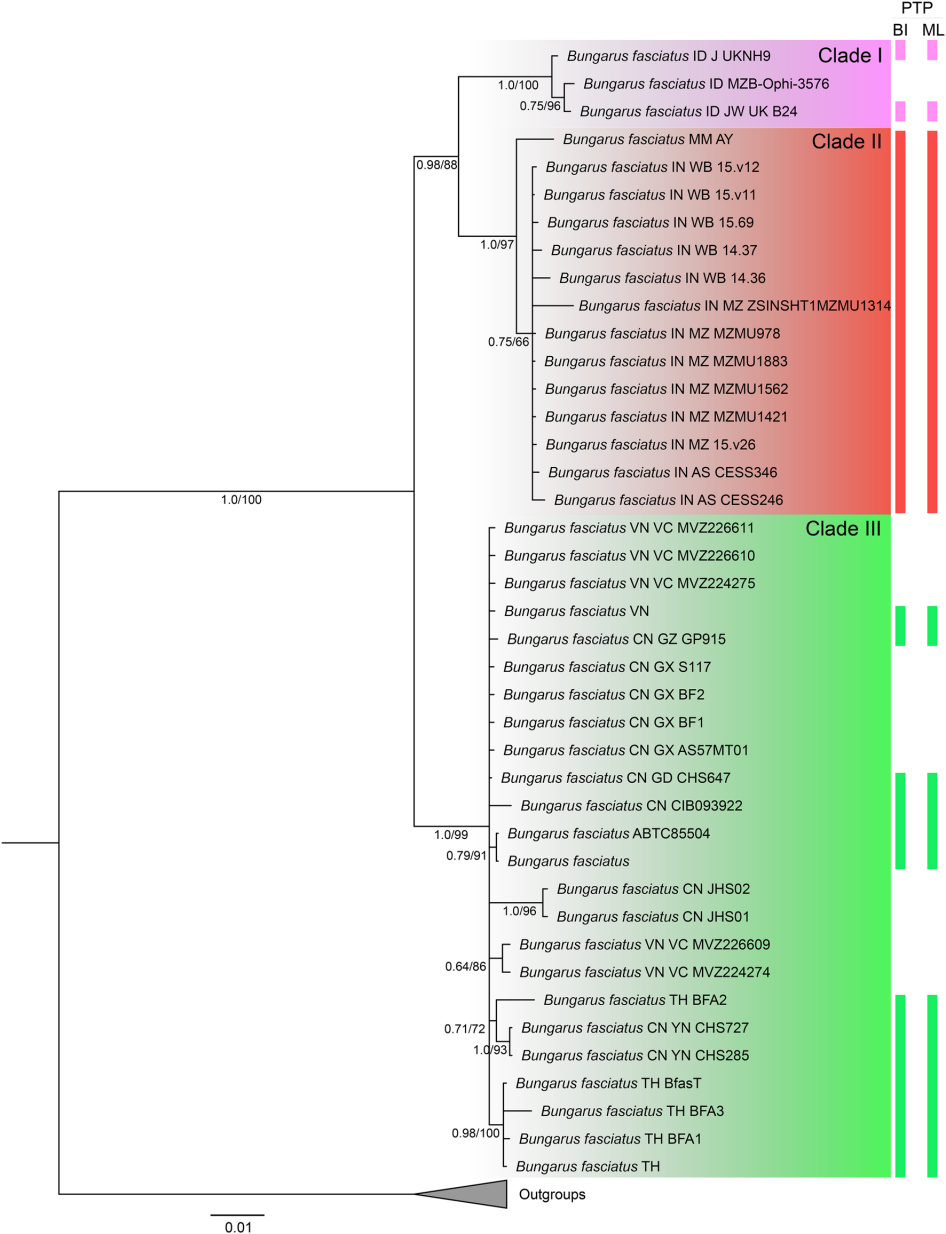
Bayesian inference (BI) phylogenetic tree based on concatenated mitochondrial 16S, COI, ND4 and CYTB genes; lineage partitions recovered from CYTB-based PTP analyses are presented besides the BI tree (only the CYTB dataset was utilized for PTP analyses because it contains more representative samples from the three clades compared to the other genes). Values at each node represent Bayesian posterior probabilities (PP) and Ultrafast Bootstrap (UFB) values from the Maximum Likelihood (ML) analysis (PP/UFB). Abbreviations of country and state/province names are: *ID* Indonesia, *JW/J* Java, *MM* Myanmar, *AY* Ayeyarwady, *IN* India, *WB* West Bengal, *MZ* Mizoram, *AS* Assam, *VN* Vietnam, *VC* Vinh Phuc, *CN* China, *GZ* Guizhou, *GX* Guangxi, *GD* Guangdong, *YN* Yunnan, *TH* Thailand.

The first 25% of trees from the BI analysis were discarded as burn-in, and the standard deviation of split frequencies were < 0.005 when analyses terminated. The graphs created using RWTY in R-Studio also indicated satisfactory topological mixing. The inferred concatenated trees from BI and ML analyses were congruent with each other. The BI tree, created using Mr.Bayes v3.2.5^65^ and further illustrated using iTOL software v5^69^, is show in Fig. 1, with Bayesian posterior probabilities from the BI analysis and UFB values from the ML analysis. The CYTB dataset consisted of a total of 1047 aligned characters, with 97 variable sites (excluding outgroups).

Molecular phylogenetic based on the concatenated aligned matrix for four mitochondrial genes (16S, COI, ND4, and CYTB; 2850 bp in length), recovered a monophyletic clade consisting of three lineages within Asia. Both the phylogenetic analyses and the single-locus-based PTP species delineation approach significantly support these three distinct clades which we describe as, (i) *B.* *fasciatus* from the Sundaic region, especially from Great Sunda islands which we describe as the Sundaic lineage (Clade I; Fig. 1); (ii) *B.* *fasciatus* from Indo-Myanmar (Clade II; Fig. 1), and (iii) *B.* *fasciatus* from mainland Sundaland including southern China, here described as east Asian lineage (Clade III; Fig. 1).

The overall mean intra-specific divergence across all lineages of *B.* *fasciatus* (uncorrected p-distance) was 3.5%. Furthermore, 0.4% intra-clade genetic divergence was observed within Clade I (between two locations in JV), 0.0%–1.3% within Clade II (between India and Myanmar), and 0.0%–6.5% within Clade III (among China, Vietnam, Thailand, and an unknown locality). The mean inter-clade genetic divergence is 5.0% between Clade I (Sundaic) and Clade II (Indo-Myanmar), 5.3% between Clade II (Indo-Myanmar) and III (east Asia); 5.7% between Clade I (Sundaic) and III (east Asia). Combined *B.* *fasciatus* (Clades I + II + III) shows the least inter-specific genetic divergence (19.5%–19.8%) with *B.* *candidus*, while inter-specific distances among other species (*B.* *sindanus*, *B.* *caeruleus*, *B.* *candidus*, *B.* *ceylonicus*, and *B.* *multicinctus*) range from 3.0% (between *B.* *candidus* and *B.* *multicinctus*) to 19.0% (between these two species and *B.* *ceylonicus*) (also see Supplementary Table S4).

### Morphometric analysis

**Figure 2:**
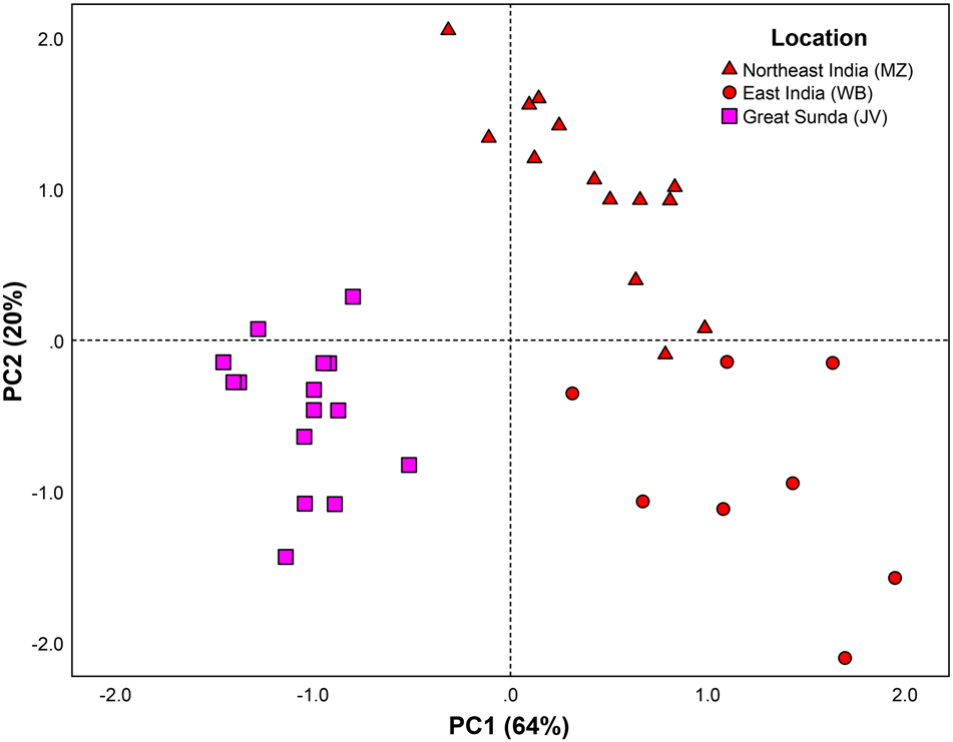
Ordination of *Bungarus* *fasciatus* populations from Mizoram (MZ), West Bengal (WB) and Java (JV) along the first two principal components based on a PCA of the characters Ve, BB, BT, and NBW. Total variance associated with PC1 and PC2 are 64% and 20%, respectively.

In this study, despite limited sampling, morphometric analyses were performed to identify taxonomically informative characters among the examined populations (WB, MZ and JV). Only the mensurals such as TaL (*p* < 0.001), HW (*p* < 0.05) and HL (*p* < 0.05) showed significantly dimorphic characters between males and females within JV and MZ populations. For meristic characters, inter-population differences were statistically significant (*p* < 0.001) for Ve (MZ vs. JV), BB, BT, and NBW (the latter three characters are tested among three populations), all of which showed a higher number in the MZ population; for mensural characters, inter-population differences were also statistically significant for TaL (*p* < 0.05) and HL (*p* < 0.001) (Table 1). Post-hoc tests conducted among the three populations for BB, BT, and NBW showed that, except for BT between MZ and WB populations (*p* > 0.05), significant differences are seen for all characters: BB (*p* < 0.001 across all the populations), NBW (*p* < 0.001 in MZ vs. WB, and JV vs. WB; *p* < 0.05 in MZ vs. JV), and BT (*p* < 0.001 in MZ vs. JV; *p* < 0.01 in JV vs. WB). Comparison was also made based on the identified variable meristic characters among the three populations using a PCA. The correlation matrix showed weak correlations between pairs of variables (r < 0.7); thus, all variables were retained for this analysis. The first two components accounted for 84% of the total variation of the data, with PC1, PC2 and PC3 representing 64%, 20% and 11%, respectively. The loadings of all variables are high on the first axis, while only Ve loads considerably highly on the second axis, with Ve having less effect on PC1 than PC2 (Supplementary Table S5). The representation of the first two components depicts substantial separation of the Javanese and the Indian populations on the first axis (PC1), and marginal separation of the WB and MZ populations on the second axis (PC2) (Fig. 2). Given that the samples from the three populations (WB, MZ and JV) were examined by different recorders, we also tested for potential recorder bias between the East Indian and northeast Indian specimens; however, no significant differences were seen after re-examination of the same specimens (*p* > 0.05).

**Table 1:** Evaluation on the meristic and mensural characters measured for 38 *Bungarus* *fasciatus* individuals from Java (JV), Mizoram (MZ), and West Bengal (WB), including mean, standard deviation, minimum and maximum values. Standardized meristic data were utilised for the following tests: Ve of JV and MZ was tested for inter-population difference and sexual dimorphism using separate one-way ANOVA with locality and sex as the factors, respectively; Sc of JV and MZ was tested using two-way ANCOVA using sex and locality as factors; BB and NBW were tested for inter-population difference (among the three populations) and sexual dimorphism (within JV and MZ) using separate one-way ANOVA with locality and sex as the factors, respectively; since BT violated the assumption of homoscedascity, it was tested using the alternative Brown-Forsythe test and was indicated by octothorp (#). For mensurals, two-way ANCOVA was performed for the log transformed TaL, HL, and HW values from JV and MZ by using the log transformed SVL as a covariate, with locality and sex as the factors. The characters with statistically significant variations at the alpha level of 0.05 are shown in boldface. The characters tested for inter-population difference across the three populations are indicated by asterisk (*).

Characters	Sex	Java (*n* = 15)		Mizoram (*n* = 15)		West Bengal (*n* = 8) unsexed		Sexual dimorphism		Inter-population difference	
		Mean ± SD	Range	Mean ± SD	Range	Mean ± SD	Range				
Ve	Male	205.44 ± 3.43	199–210	226 ± 2.10	222–228	217.63 ± 3.12	212–222	*F*_1,28_ = 1.35	*p* = 0.256	*F*_1,28_ = 469.80	*p* < **0.001**
	Female	206.83 ± 1.94	205–210	229.11 ± 2.15	224–231						
Sc	Male	34.43 ± 0.98	33–36	35.83 ± 0.75	35–37	34.63 ± 1.49	31–36	*F*_1,25_ = 2.44	*p* = 0.131	*F*_1,25_ = 1.30	*p*** = **0.266
	Female	31.17 ± 1.60	30–34	33.75 ± 1.28	32–36						
BB	Male	22.67 ± 1.12	21–25	24.33 ± 1.97	22–27	28.38 ± 1.73	26–31	*F*_1,28_ = 0.44	*p* = 0.511	*F*_2,35_ = 39.78*	*p* < 0.001*
	Female	21.83 ± 1.17	20–23	25.00 ± 1.58	23–27						
BT	Male	3.22 ± 0.67	2–4	5.00 ± 0.00	5	5.25 ± 1.09	4–7	*F*_1,21_ = 0.12^#^	*p* = 0.728^#^	*F*_2,12_ = 17.86*^#^	*p* < 0.001*^#^
	Female	3.17 ± 0.41	3–4	4.22 ± 0.44	4–5						
NBW	Male	19.00 ± 1.00	18–20	18.20 ± 0.45	18–19	15.63 ± 1.11	14–17	*F*_1,27_ = 0.40	*p* = 0.533	*F*_2,34_ = 22.16*	*p* < 0.001*
	Female	19.00 ± 0.63	18–20	17.67 ± 1.73	15–20						
TaL	Male	120.74 ± 20.01	90–145	101 ± 38.92	47–133	–	–	*F*_1,24_ = 18.96	*p* < **0.001**	*F*_1,24_ = 6.01	*p* = **0.022**
	Female	107.86 ± 23.43	85–145	97.88 ± 15.56	76–119						
HL	Male	35.06 ± 4.97	27.10–40.90	21.60 ± 5.71	12.80–26.60	–	–	*F*_1,24_ = 4.37	*p* = **0.047**	*F*_1,24_ = 79.38	*p* < **0.001**
	Female	34.81 ± 6.19	25.90–44.50	21.03 ± 5.03	15.74–29.68						
HW	Male	20.88 ± 4.03	13.80–25.70	17.79 ± 5.10	12.18–22.46	–	–	*F*_1,25_ = 4.33	*p* = **0.048**	*F*_1,25_ = 0.97	*p*** = **0.334
	Female	20.70 ± 3.13	16.40–26.20	16.12 ± 4.30	10.40–22.76						

Significant values are in bold.

### Systematics

We present diagnostic morphological, morphometric, and meristic data taken for *Bungarus* *fasciatus* Clade II from east and northeast India (Supplementary Table S6). The examined specimens of *B.* *fasciatus* from India are morphologically distinguishable from the Sundaic population (see Table 2). Based on the present study, we postulate the existence of at least three different taxonomic entities within the nomen *B.* *fasciatus*, and also confirm that populations in eastern India (e.g. Odisha, WB, etc.) and northeastern India (e.g. MZ, Assam, etc.) are conspecific. Based on the original description of *Pseudoboa* *fasciata*, minimum three specimens were available or referable to Schneider^82^; hence syntypes. Among these syntypes two specimens (ZMB 2771, 2772) have been deposited at ZMB from the collection of Marcus Bloch (fide Bauer^83^). In addition, one of syntypes was depicted in Russell^84^ (page 3, plate 3) as the “Bungarum Pamah”, an adult from “Mansoor Cottah” (now Gobalpur, Odisha (Orissa), India), specimen is now lost (fide Bauer^85^). So far, the only existing name-bearing type specimens are the two syntypes in the collection of Berlin Zoological Museum (ZMB 2771–72) originating from “Indien” (= India) fide ZMB catalogue^36^ a detailed taxonomic revision will be published elsewhere (Amarasinghe et al. in preparation). We affirm that the specimen used by Russell^84^ for his illustration is the same specimen (syntype) housed in the ZMB, thus we adhere with the type locality given by Russell^84^. Therefore, here we postulate the Indo-Myanmar populations (Clade II) as *B.* *fasciatus* sensu stricto, while considering the populations from Sundaic region, especially from Greater Sunda Islands (Clade I) and mainland Sundaland including southern China (Clade III) as *B.* *fasciatus* sensu lato. Consequently, we redescribe the *B.* *fasciatus* sensu stricto, including hemipenis morphology, based on MZ population, from where a large number of samples are available.

**Table 2:** Some comparative morphological data of *Bungarus* *fasciatus* sensu lato in each biogeographic region, based on this study and published data.

Character	Population/clade		
	Indo-Myanmar (*n* = 23)	East Asia (*n* = 11)	Greater Sunda (*n* = 15)
Ventrals	200–234	217–237	199–210
Subcaudals	23–39	33–41	30–36
Number of dorsal bands on body	22–31	19–21	20–25
Number of dorsal bands on tail	4–7	?	2–4
Nuchal band covered by vertebral scales	14–20	?	18–20
Background body color	Yellow/cream	Yellow	Yellow/cream
Source	Smith^75^ This study	Yang & Rao^76^; Chen et al.^54^; Leviton et al.^77^	This study

### *Bungarus fasciatus* (Schneider, 1801) sensu stricto

**Figure 3:**
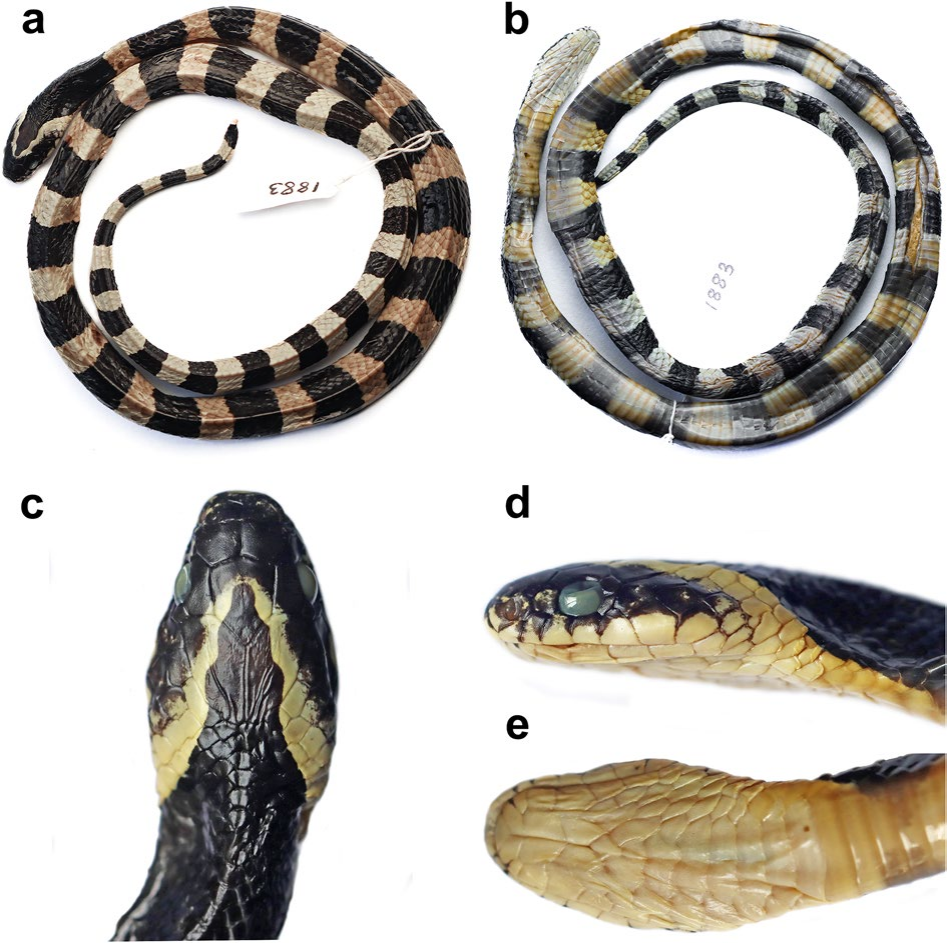
*Bungarus* *fasciatus* sensu stricto (MZMU1883) from Northeast India: (**a**) dorsal view of full body, (**b**) ventral view of full body, (**c**) dorsal view of head, (**d**) lateral view of the left side of head, and (**e**) ventral view of head.

**Figure 4:**
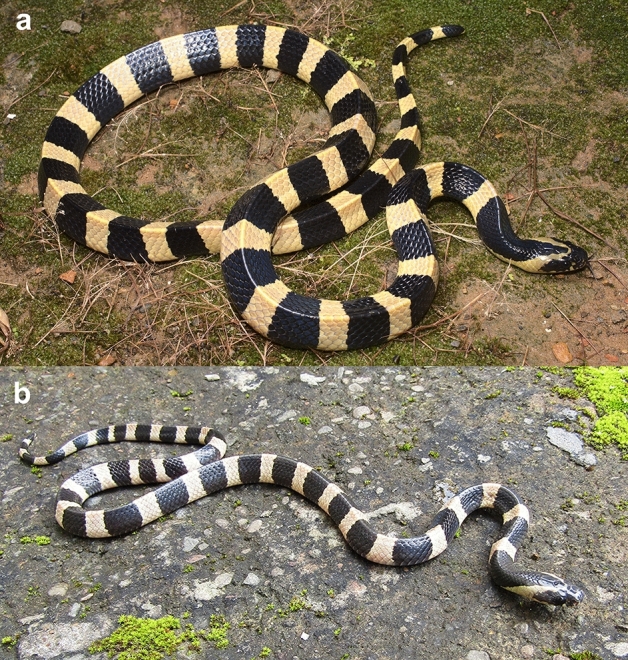
Live individuals of *Bungarus* *fasciatus* sensu stricto (**a**) from Keitum village, Mizoram, India (MZMU1421), and (**b**) a juvenile with creamish dorsum coloration from Saikhawthlir village, Mizoram, India.

**Figure 5:**
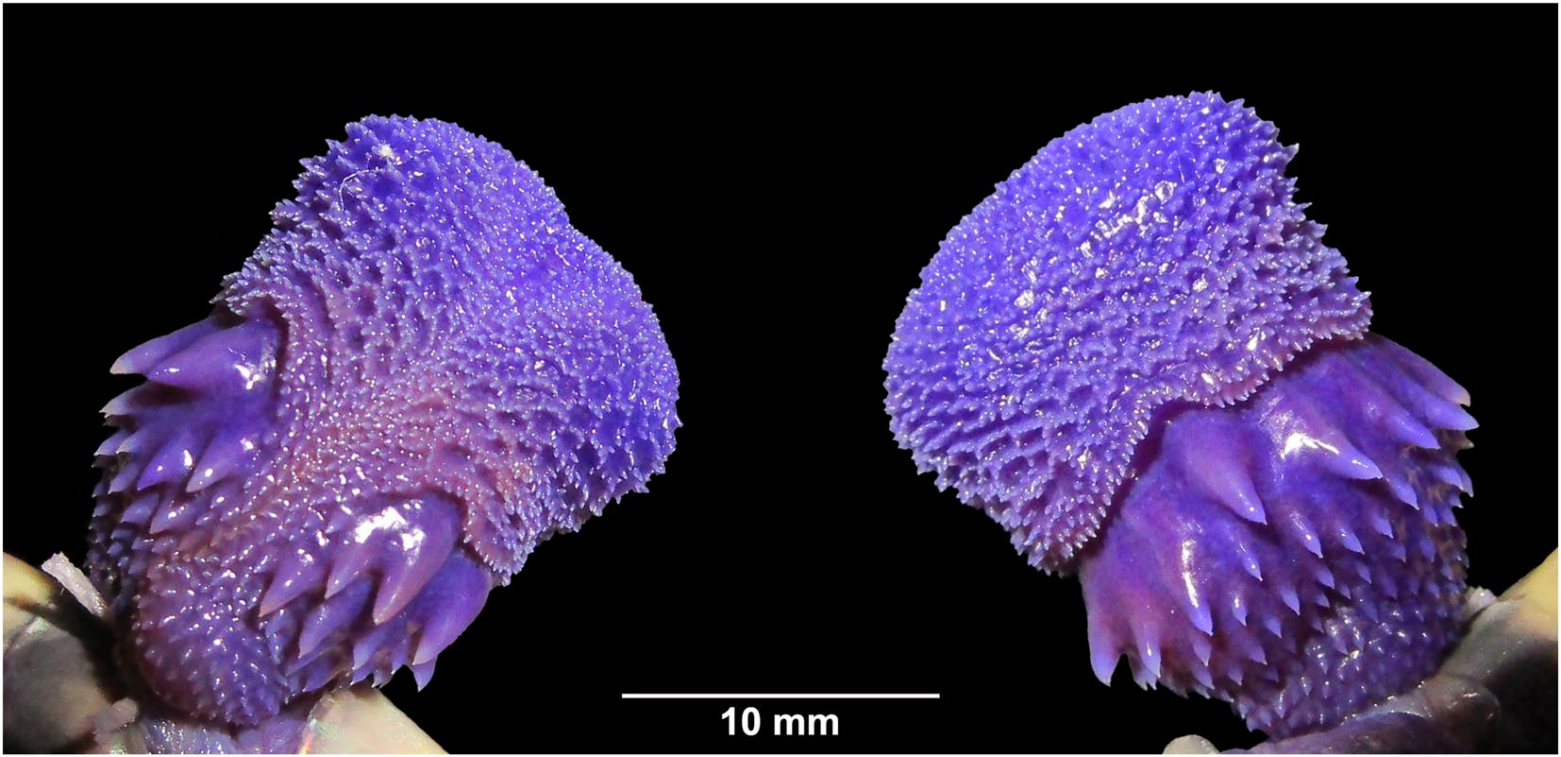
Sulcal (left) and asulcal (right) views of the right hemipenis of *Bungarus* *fasciatus* sensu stricto (MZMU2935) from Mizoram, India.

(Tables 1, 2; Figs. 3A–E, 4A,B, 5).

[English: Banded krait; Bengali: Sankhamuti/Sankhini/Chamorkasa; Mizo: Chawnglei/Tiangsir].

*Pseudoboa* *fasciata* Schneider, 1801.

*Bungarus* *annularis* Daudin, 1803.

*Bungarus* *fasciatus* *bifasciatus* Mell, 1929.

*Bungarus* *fasciatus* *insularis* Mell, 1930.

### Examined materials

Males (*n* = 7; MZMU 933, 1314, 1320, 1417, 1421, 1883, 2935) and Females (*n* = 8; MZMU 1319, 1321, 1550, 1562, 1561, 1548, 1572, 2481) collected from MZ, northeast India.

### Species redescription

Based on the overall examined MZ materials with combined sexes, adults SVL 444.0–1220.0 mm, tail length 47.0–133.0 mm; head elongate (HL 2.0–3.5% of SVL), wide (HW 71.8–92.1% of HL), slightly flattened, indistinct from neck; snout elongate (ES 22.8–40.1% of HL), moderate, flat in dorsal view, rounded in lateral profile, rather depressed. Rostral shield large, flat, slightly visible from above, pointed posteriorly; interorbital width broad; internasals subtriangular; nostrils rather large, nasals large, divided, and elongated, in anterior contact with rostral, and internasal and prefrontal dorsally, 1st and 2nd supralabial ventrally, preocular posteriorly; no loreal; prefrontal rather large, broader than long, and pentagonal; frontal large, hexagonal, short, slightly longer than width; supraoculars narrow, elongate, subrectangular, posteriorly wider; parietals large, elongate, butterfly wing-like in shape, bordered by supraoculars, frontal, upper postocular anteriorly, anterior and upper posterior temporals, and five or six nuchal scales posteriorly; one preocular, vertically slightly elongated, hexagonal, in contact with prefrontal and posterior nasal anteriorly, supraocular dorsally, and 2nd and 3rd supralabials ventrally; eye moderate (ED 10.7–21.7% of HL), round, about half of the size of snout length (ED 41.7–69.9% of ES), pupil rounded; two postoculars, subequal or upper one larger, pentagonal, upper postocular in broad contact with supraocular, parietal and anterior temporal, lower postocular in contact with anterior temporal and 5th supralabials; temporals 1 + 2, large, slightly elongated, subrectangular or pentagonal; anterior temporal larger than posterior temporal, in contact with parietal and both postoculars dorsally, and 5th and 6th supralabial ventrally; lower posterior temporal in contact with 6th and 7th supralabials ventrally. Supralabials seven (on both sides), 5th–7th largest in size; 1st supralabial in contact with rostral anteriorly, nasals dorsally, 2nd with posterior nasal and preocular dorsally, 3rd with preocular and orbit dorsally, 4th with orbit; 5th with orbit, lower postocular, and anterior temporal dorsally, and 6th with anterior and lower posterior temporals dorsally, 7th with lower posterior temporal dorsally and scales of the neck posteriorly.

Mental large, triangular, blunt posteriorly; first infralabial pair larger than mental plate and in broad contact with each other, in contact with anterior chin shields posteriorly; seven infralabials, 1st–4th in contact with anterior chin shields, 4th infralabial largest in size in contact with both anterior and posterior chin shields; 4th–7th infralabials in contact with gular scales; two larger anterior chin shields, and two slightly smaller posterior chin shields; anterior chin shields in broad contact between them; posterior chin shields bordered posteriorly by seven gular scales.

Body robust, elongate and subcylindrical; dorsal scales in 15 midbody rows, all smooth and pointed posteriorly; 222–228 ventrals in males and 224–231 in females; cloacal plate divided. Tail comparatively short, TaL 8.9–10.4% of total length in males and 13.5–17.1% of total length in males, robust and thick; subcaudals 35–37 in males and 32–36 in females, divided.

### Coloration

In preservative, dorsum and venter white or yellow; 22–27 black cross bands along the body and 4 or 6 on the tail; cross bands complete laterally, and reaching the ventrals except the nuchal band; the bands on the tail distinct; the nuchal band on the nape anteriorly inverted V-shaped covering 15–20 vertebral scales; nuchal band starts from mid frontal; snout, anterior head, and lateral head black making remaining the white dorsal color an inverted V-shaped marking; first black band on the body covering 6 or 7 vertebral scales; inter-band width covers with 3–5 vertebral scales; lower parts of the supralabials white; ventral head white until the first black band; tail tip black dorsally, white ventrally.

In life (Fig. 4A), same color as in preservative, but the white body color may vary from white, cream, pale yellow to bright yellow. One juvenile with cream and black body bands was encountered in Saikhawthlir, MZ (Fig. 4B), but the snake escaped before recording morphological data.

### Variation

Except the anomalous specimen (MZMU1321) which had three postoculars on left and two on right, and temporals 1 + 2 on the left and 2 + 2 on the right, all other meristic and morphometric characters obtained so far did not show any significant variation between the examined populations, and also correspond to the conventional taxonomical characters provided in previously published literature^77,86,87^.

### Hemipenis

Based on MZMU2935, the organ is single and subcylindrical, relatively short, robust, and capitate; inverted hemipenis extends to 4th–7th subcaudal level (i.e. 11.1–20% from the total number of Sc); sulcus spermaticus bifurcate below the crotch, shallow and centripetal; apical lobe less evident with only slight apical flaring; calyculate organ with a complex ornamentation of retiform ridges, papillate flounces, and spines; spines on the upper basal areas enlarged and decreasing the size towards the proximal portion; apical region sharply separated from the basal portion by a well-defined demarcation, so the apex is free and the apical part of the hemipenis is richly capitate (Fig. 5).

### Distribution

Within India, *B.* *fasciatus* has been reported from Uttar Pradesh (Gorakhpur, fide Masson^88^; also see Anwar^89^ and Das et al.^90^) in the north and central Maharashtra in the west^91–93^, extending across Telangana (Hyderabad, fide Kinnear^94^, Andhra Pradesh^95^, Chhattisgarh^96,97^, Jharkhand (Koderma, fide Smith^86^; also see Husain^98^), Bihar^99^, Odisha (Mahanadi valley, fide Wall^99^; also see Boruah et al.^100^), and northern part of WB^101^ to northeastern India, including Arunachal Pradesh^102,103^, Assam^99,104,105^, Meghalaya^106^, MZ^107,108^, Tripura^109^, Manipur^110^ and Nagaland^111^. A few unverified records are available from Madhya Pradesh^36^, Uttarakhand^35^, and southern peninsular India in Tamil Nadu, Karnataka and Kerala^98^.

**Figure 6:**
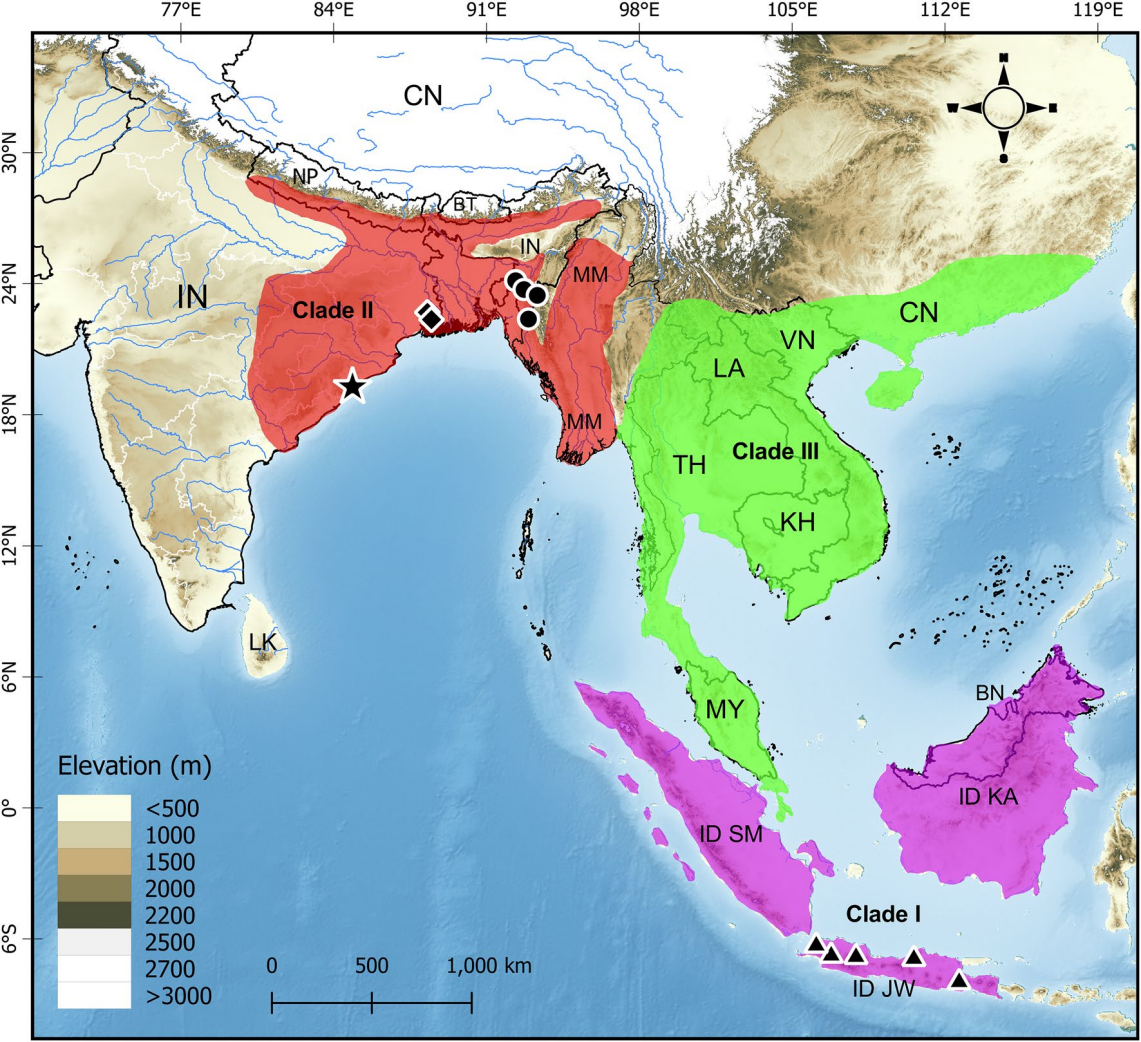
Map showing the distribution range of *Bungarus* *fasciatus* sensu lato*,* based on the latest species map provided by the World Health Organization (2022); the coloration corresponds to the three distinct evolutionary lineages recovered in the phylogenetic analyses. The type locality of *Bungarus* *fasciatus* sensu stricto is indicated by a black star. Localities of specimens used in the morphological analyses are indicated by black filled diamonds (WB), circles (MZ), and triangles (JV). Abbreviations for countries are: *IN* India, *NP* Nepal, *BT* Bhutan, *BD* Bangladesh, *LK* Sri Lanka, *CN* China, *MM* Myanmar, *LA* Laos, *TH* Thailand, *VN* Vietnam, *KH* Cambodia, *MY* Malaysia, *BN* Brunei Darussalam, *ID* Indonesia (*KA* Kalimantan, *SM* Sumatra, *JW* Java).

Here we provide additional distributional records for *B.* *fasciatus* sensu stricto based on 44 new localities from MZ, and two from WB, India (Supplementary Table S7). The lowest elevation among these new records is 4 m a.s.l. at Chitrasali in Hooghly District, WB and the highest is 1426 m a.s.l. at Champhai Jailveng in Champhai District, MZ. Based on the previous distribution of the species, the elevation range was between 40 and 2300 m a.s.l.^33,34^. Moreover, an estimated distribution range of the species was plotted (Fig. 6) following WHO’s range estimation for *B.* *fasciatus*^112^.

### Natural history

Although *B.* *fasciatus* is a common species, details on the ecology, habitat, population, and breeding are still sparse and further studies are needed. Therefore, here we provide some natural history data based on two clutches of eggs encountered from two localities in WB State, India:

(i) On 16th May 2020, at ca. 20:00 h, from Chitrasali village, Hooghly, the snake was encountered on the bank of a pond adjacent to a house in the middle of a village. The female was found coiling around a clutch of 19 eggs. The breeding site was located inside a naturally occurring burrow at the base of a dead tree with decayed roots. The burrow was on the bank ca. 6 feet from the pond. The pond had a gentle slope and was surrounded by plentiful vegetation. On the day of the egg collection, the recorded ambient temperature at the natural breeding site was 28–38 °C with average humidity of 78%. The eggs were relocated and incubated in a dedicated herpetoculture room at 27.6 °C using 3 cm thick vermiculite bedding in a perforated box. On 10th June at 20:18 h, the first egg slits were observed, and hatching was completed on 18th June at 05:45 h. The fluctuating room temperature and average humidity from the start of hatching until hatching was completed were 26–35 °C and 81.1%, respectively. Notably, hatchlings crawled out from the pipped eggs on the 12th, 13th, and 14th June. Upon investigation, we found that a total of six eggs failed to hatch, out of which three eggs were unfertilized, two contained partially developed embryos showing deformities, and one egg had a fully developed embryo, possibly unable to cut through the eggshell. On 18th June, we recorded the biometric data of the 13 hatchlings (5 females with average SVL 322.2 mm, TaL 32.4 mm, and body weight 21.2 g; 8 males with average SVL 318.6 mm, TaL 36.5, body weight 19.9 g), and were subsequently released close to where the eggs were collected.

(ii) On 05th May 2021 at 12:30 pm, from a construction site at Ankuni village, Hooghly. A clutch of eight eggs were uncovered under a pile of old bricks at the base of a dead tree with lots of burrows. The breeding site was located on the bank of a pond, and the entire rubble pile was covered in vegetation. However, in this case, the female snake was not found near the eggs, and it is possible that the excavation work might have scared the female away. The eggs were relocated and incubated in the same herpetoculture room using 3 cm thick vermiculite bedding in a perforated box. The room temperature recorded on 5th May fluctuated between 24 and 33 °C, with a relative humidity of 65%. Egg slits were seen on 6th June at ca. 22:00 h. On 8th June at ca. 08:00 h, hatching was completed and all of the juveniles had emerged from the eggs. From the egg relocation until the completed hatching (6th–8th June), the temperature and humidity fluctuated between 24 and 39 °C and 65–75%, respectively. On 8th June, the biometric data of the eight hatchlings were taken (3 females with average SVL 333.3 mm, TaL 38.7 mm, and body weight 21.3 g; 5 males with average SVL 351.0 mm, TaL 43.2, body weight 21.4 g), and they were also released close to the site from which the eggs had been collected.

## Discussion

### *Bungarus fasciatus* sensu stricto

Evidence from this study, based on morphology and molecular data, defines three distinct clades of *B.* *fasciatus* with non-overlapping distribution clusters. The high genetic divergence among lineages also suggests distinct species-level groups within *B.* *fasciatus* as currently conceived. Our morphometric data analysis also provides evidence of their morphological distinctiveness between Clade I and II. Moreover, the lineage from east Asia is basal to the other two lineages but, if these clades were to be accepted as full species, the name-bearing lineage is Clade II. Thus, according to our newly presented evidence, and partly according to Russell^84^, the distribution range of *Bungarus* *fasciatus* sensu stricto (Indo-Myanmar clade) comprises east and northeast India extending towards Myanmar. (Figs. 1, 6)*.*

### Systematic challenges

In this study, we elucidate the presence of three independent lineages within *B.* *fasciatus,* which is crucial for future nomenclatural revision. In the CYTB gene, while negligible intra-clade genetic divergence was observed within Clade I (0.4%; between two locations in JV) and Clade II (0.0–1.3%; Myanmar, east and northeast India), a wide range of intra-clade genetic divergence (00.0–6.5%) was evident within Clade III (China, Vietnam, Thailand). Consequently, we speculate that there might still be cryptic diversity within the east Asian lineage (Clade III). Moreover, for robust delimitation of the *B.* *fasciatus* complex, it is necessary to establish whether these lineages have undergone some degree of extrinsic or intrinsic reproductive isolation to be evolving separately^113^. For instance, due to the high evolutionary rate of hemipenial traits compared to the other morphological traits^114,115^, the organ has commonly been used to provide a picture of sexual barrier even among cryptic species^116–118^.

Although it has been previously stressed that delimiting the taxonomic status of geographically diversified populations of venomous snakes alone cannot necessarily predict patterns of venom variation, it can play a pivotal role in overcoming the consequential variability of venoms^119–121^. Fry et al.^120^ further indicated that the medical importance of *B.* *fasciatus* has been overestimated. Moreover, the possible existence of undiscovered cryptic species accompanied by more venom diversity with uncharacterized components had been pointed out^122^. Siqueira-Silva et al.^123^ observed that more productive environments favour more complex venom, with more toxins in similar proportions. Based on the verbal autopsy we have conducted so far within MZ, there are three cases of fatal envenomation potentially from the bite of banded krait. Therefore, here we highlight the importance of analyzing the venom compositions in different populations in each biogeographically isolated clade.

### Further work

The combination of multivariate morphometric analysis and mitochondrial gene-based phylogeography has been applied successfully for species delineation^24,124,125^ as well as for testing species boundaries^126^. However, nuclear genes provide an independent test of species boundaries^127^ as they are capable of measuring the extent of gene flow, and for this reason, recent work has increasingly used a combination of nuclear and mitochondrial genes for phylogeographic analyses and species delineation^128^. Consequently, we believe that the potentially species-level diversity across different *B.* *fasciatus* populations depicted in this study cannot be overlooked, and a thorough comprehension of *B.* *fasciatus* systematics is still a fundamental challenge.

## Supplementary Information

Supplementary Table S1.

Supplementary Table S2.

Supplementary Table S3.

Supplementary Table S4.

Supplementary Table S5.

Supplementary Table S6.

Supplementary Table S7.

## Data Availability

The generated partial gene sequences were deposited on the NCBI repository (GenBank accession numbers are given in Supplementary Table S2).
